# Spontaneous Activation of Event Details in Episodic Future Simulation

**DOI:** 10.3389/fpsyg.2019.00625

**Published:** 2019-03-21

**Authors:** Yuichi Ito, Yuri Terasawa, Satoshi Umeda, Jun Kawaguchi

**Affiliations:** ^1^Department of Psychology, Keio University, Tokyo, Japan; ^2^Japan Society for Promotion of Science, Kojimachi Business Center Building, Tokyo, Japan; ^3^Department of Cognitive and Psychological Sciences, Graduate School of Informatics, Nagoya University, Nagoya, Japan

**Keywords:** episodic future thinking, future simulation, prospective memory, intention superiority effect, contextsensitivity, memory retrieval, recognition

## Abstract

Episodic future simulation is supported by both the retrieval and recombination of episodic details. It remains unclear, however, how individuals retrieve episodic details from memory to construct possible future scenarios; for this people must use details related to the planned future events appropriately. A potentially relevant cognitive process is the spontaneous activation of intention observed in prospective memory (i.e., the intention superiority effect). Previous studies on prospective memory have shown that the approximation of retrieval opportunities for future intentions activate related information, suggesting that the intention superiority effect is context-sensitive. We hypothesized that the same cognitive process underlies future simulation—that is, details related to future events should spontaneously become activated at the appropriate moment of future simulation to make that simulation plausible. In Experiment 1, participants took part in future experiments and formed intentions to perform particular actions for the next experiments. Subsequently, they imagined events that could occur up until they arrived at the experimental room on the day of the next experiment. During this exercise, they did not imagine engaging in the required experimental task. We measured the conceptual activation of intention-related information via a recognition task using intended action words as targets. The results showed the intention superiority effect—concepts related to participants’ future intentions became active when envisioning future events approaching the next experiment. In Experiments 2 and 3, we ensured that the intention superiority effect in future simulation was context-sensitive by adding a control condition that required participants to imagine events other than the approaching future experiments. These results indicated that concepts related to the intended actions were spontaneously activated when imagined future events became both temporally and spatially close to the future simulation. Our finding suggests that spontaneous activation of details approaching the context of a future simulation helps in constructing plausible future scenarios.

## Introduction

Episodic future simulation is the ability to mentally project oneself into possible future events and thereby “pre-experience” them (Atance and O’Neill, 2001; Atance and O’Neill, 2005; Schacter et al., 2007). It is an exceedingly adaptive and important mental activity in our daily lives (D’Argembeau et al., 2011; Schacter, 2012). Future simulation, according to the “constructive episodic simulation hypothesis,” is supported by both the retrieval and recombination of episodic details (Schacter and Addis, 2007; Schacter et al., 2007, 2008). There is plenty of research in support of this hypothesis. However, it remains unclear how individuals retrieve episodic details from memory to construct “plausible” future scenarios. To explore this, we focused on spontaneous memory activation, a background process of prospective memory, as a retrieval process aiding in the construction of plausible future events.

Research on the retrieval process of episodic future simulation has shown that it often uses primed information (Szpunar, 2010), and that people can, to some extent, inhibit certain parts of retrieved memories for episodic future simulation (Ditta and Storm, 2016). The former study described the semantic priming effect on future thinking and highlighted how there is a processing pathway by which people retrieve conceptually activated information and use it to construct future scenarios. The latter study, in contrast, found support for memory inhibition caused by retrieval (Anderson et al., 1994). Both studies described the effect of unintentional memory retrieval processing in future simulation. With regard to the semantic priming effect, people who experience some degree of priming tend to always think about information related to the prime (Szpunar, 2010), which implies that people do not always construct future scenarios simply in accordance with their level of conceptual activation; rather, they seem to select the most helpful information for constructing plausible future thoughts from memory. An alternate interpretation for this finding is that there may also be priority levels among retrieved details when constructing scenarios that people will possibly experience in the future, according to the context of the imagined events. For instance, when an individual is planning for an academic conference tomorrow, they are more likely to imagine an academic situation in constructing future simulations of that event even when primed by leisure-related information. By contrast, using leisure-primed information to simulate problems with travel might not result in plausible future scenarios.

In typical paradigms for studying episodic future thinking, participants are asked to envision events that are plausible to occur in the future (D’Argembeau and Van der Linden, 2004; Addis and Schacter, 2008; Arnold et al., 2011a). People need to appropriately consider details related to almost fixed plans for future simulations (e.g., time or place of implementing intention). If people are unable to use such information, they are likely to fail in imagining “plausible” future events. Thus, there is likely a cognitive process by which information contextually associated with imagined future events is accessed. In this study, we hypothesize that this process is the spontaneous activation of details during future simulation, a phenomenon observed in prospective memory tasks.

Prospective memory is the spontaneous recall of memories relevant to an intended action at an appropriate timing in the future (Goschke and Kuhl, 1993; Marsh et al., 1998; McDaniel and Einstein, 2007). An important feature of prospective memory is the “intention superiority effect,” which refers to a heightening of accessibility of intention-related concepts depending on the proximity of a context relevant to that intention (Sellen et al., 1997; Schult and Steffens, 2013). This effect ensures that people do not forget to perform intended actions in an appropriate future moment. For example, if an individual intends to take medicine after a meal, they may spontaneously be reminded of this when the meal is finished. In the experiment of Schult and Steffens (2013), all participants were asked to learn two lists of actions and were told that they would have to perform either of these lists later. Subsequently, half of them were informed that the action performance task would come just after a recognition task requiring them to judge whether presented words had been in the studied lists or not. This manipulation ensured that the retrieval opportunity would be close at hand. The other half of the participants were informed that the performance task would be conducted after another task following recognition task (i.e., the retrieval opportunity was not close). They subsequently found that, during the recognition task, participants showed faster recognition of action-list words than non-action-list words only in the proximal-retrieval condition. Together, these results suggest that intention-related concepts spontaneously activate due to the proximity of intention-related retrieval opportunities; in other words, the intention superiority effect is context-sensitive.

We believed that this context-sensitivity of the intention superiority effect could also be applied to episodic future simulation. To engage in such future-oriented mental time travel (Schacter et al., 2007) that has real-world applicability, people must construct a relatively accurate representation of the temporal sequence of events pertaining to the simulation. Even when a retrieval opportunity is distant, people can project themselves into the situations leading up to that retrieval opportunity during mental simulation. Potentially, the context-sensitive spontaneous activation of intention-related concepts (i.e., intention superior effect) may arise as individuals’ mental travel nears the simulated retrieval opportunity, providing individuals with the necessary details to construct plausible future scenarios.

In the present study, we conducted three experiments using the paradigm of Schult and Steffens (2013). Although their experiment captured conceptual activation of intended actions due to the proximity of a retrieval opportunity in the real world, our experiments captured such activation due to the proximity of mentally simulated retrieval opportunities. In Experiment 1, we investigated whether the intention superiority effect arises during future simulation, while in Experiments 2 and 3, we confirmed the context-sensitivity of the intention superiority effect for future simulation by adding a condition requiring participants to imagine another future scenario approaching the performance task (i.e., approaching condition vs. another-day condition). We expected that participants’ recognition time would be shorter and their recognition performance better in Experiment 1 and in the approaching condition of Experiments 2 and 3, if information related to the intended actions is activated by participants’ proximity to the retrieval opportunity (i.e., the performance task).

## Experiment 1

We first investigated whether the intention superiority effect is observed in episodic future simulation. According to a previous study (Schult and Steffens, 2013), the effect does not arise for temporally distant intentions. However, the effect should occur while mentally getting close to the retrieval opportunity by future simulation.

### Methods

#### Participant

Twenty-six undergraduate students of Nagoya University participated in Experiment 1 (*M*_age_ = 18.81, *SD*_age_ = 0.49). There were 11 males and 15 females. The ethics committee of the Graduate School of Environmental Studies at Nagoya University approved this study. All subjects gave written informed consent in accordance with the Declaration of Helsinki. One participant, who had no experience of brewing coffee (one of the simulated actions) or seeing such an action, was excluded from analysis. Ultimately, the data from 25 participants were analyzed.

#### Stimulus

Two action lists (i.e., brewing coffee and making a sandwich) were created on the basis of previous studies (Goschke and Kuhl, 1993; Schult and Steffens, 2013). The actions for brewing coffee were “placing the filter,” “pouring hot water,” “measuring the coffee,” “melting the sugar,” and “stirring in the milk.” The actions for making a sandwich were “roasting bacon,” “tearing lettuce,” “toasting bread,” “spreading mayonnaise,” and “putting in sandwich fillings.” Each list comprised 5 actions combining a noun and a verb. The stimuli of both study lists were comparable with regard to word length [*M* = 2.90, *SD* = 1.10 for brewing coffee and *M* = 2.70, *SD* = 1.25 for making a sandwich; *t*(18) = 0.379, *p* = 0.709, Cohen’s *d* = 0.163] and word frequency [*M* = 7380.70, *SD* = 13077.58 for brewing coffee and *M* = 7412.40, *SD* = 18562.66 for making a sandwich, according to Amano and Kondo, 1999; *t*(18) < 0.01, *p* = 0.99, Cohen’s *d* < 0.01). The 10 nouns and verbs used in these two lists were included in the recognition task, along with 10 further nouns and verbs as distractor words (see Supplementary Table S1).

#### Procedure

**Figure 1:**
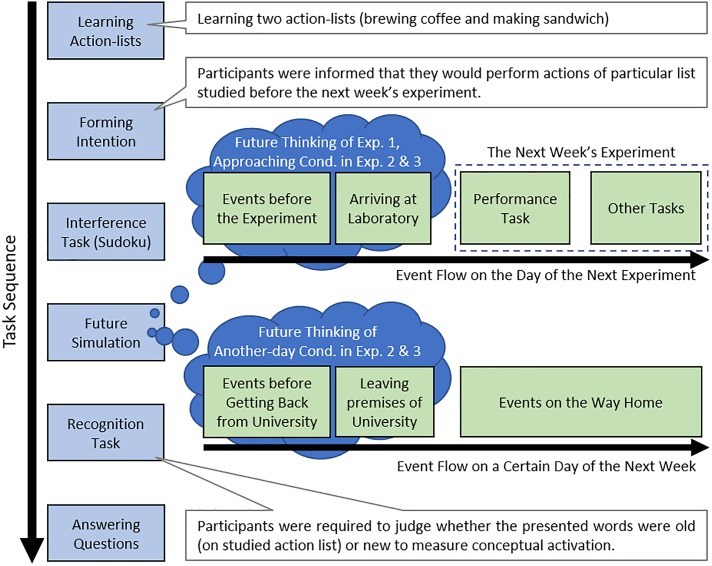
Schematic representation of the procedure of the present experiments. The procedure was the same in Experiments 1 and 2 except for the future simulation phase. In Experiment 1 and the approaching condition of Experiments 2 and 3, participants imagined future events until they arrived at the experimental room. In the another-day condition of Experiments 2 and 3, participants imagined future events from when they left the university ground to returning home.

Although the experiment was conducted as a one-shot, all participants were recruited under the pretext that the experiment would consist of two sessions in 2 weeks. The experiment proceeded as follows: (1) learning action lists, (2) forming intentions, (3) interference task, (4) future simulation task, (5) recognition task, and (6) questionnaire (Figure 1). The stimulus presentation and data collection were controlled by E-prime 2.0 (Psychology Software Tools, Inc.).

First, when participants entered the experimental room, they were asked to schedule a second experimental day in the next week. After that, participants were required to memorize two action lists (i.e., the brewing coffee and making a sandwich lists). The coffee list was named “list 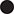
” and the sandwich list was named “list 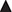.” The actions in each list were presented serially in a fixed order with corresponding list name; it means that the list name was presented in the top of the display, and an action was presented in the center of the display. Each action was presented for 5000 ms following a 250-ms fixation point and 250-ms blank screen (in that order). The inter-trial intervals were 500 ms. The lists were alternately presented three times each, and the order of the two lists was counterbalanced across participants.

After learning the action lists, participants were told, “When you enter the experimental room for the session in the following week, immediately go to the next room, which has a kitchen, and perform either of the two action lists. After this performance task, you will return to this room and participate in some cognitive tasks.” Subsequently, they were informed that they would engage in three tasks: a memory task, number puzzle task (i.e., Sudoku), and future simulation task; the tasks would be given in a random order and controlled by a computer (however, the task order was actually fixed). The participants were given a brief description of each task to ensure that they smoothly progressed through the rest of the experiment. The instructions were presented on the display and were read aloud by the experimenter.

Subsequently, participants were given the action list for the next experimental session. Half the participants were assigned to the coffee list and the other half to the sandwich list. At that time, we emphasized that we would not inform them of the performance list to be executed in the next session. Participants then immediately engaged in the number puzzle task for 1 min (to prevent rehearsal of the performance list while keeping their intention accessible in working memory). For this task, participants were given Sudoku sheets and pens when the computer screen shifted to the instructions of the number puzzle task. The experimenter verbally confirmed if participants understood the rules of Sudoku, after which they pressed a key to start the task. The screen displayed instructions to “perform the number puzzle” for 1 min, after which it displayed “stop the number puzzle task.”

The screen then automatically shifted to instructions for the future simulation task, which reiterated the brief description of the tasks. Participants were told that, for approximately 3 min (and always more than 2 min), they should mentally simulate and verbally report events that had occurred up until they arrived at the experimental room on the day of the session in the following week; they had to stop imagining the moment when they mentally arrive at the experimental room. They were instructed to imagine specific events (i.e., unique to a time and place) as if they were pre-experiencing those events. Events could not be longer than one day—they had to imagine events occurring from the time they woke up till the time they entered the experimental room on the specific day. Participants envisioned the future for approximately 3 min; after 2 min had passed, the experimenter informed the participants of the time. Participants pressed a key to finish the task at the moment that they entered the experimental room in the future simulation scenario. This was followed by an instruction for participants to end the future simulation task.

Subsequently, the screen automatically changed to display the instructions for the recognition task. In the recognition task, participants were told that they would judge whether words had appeared in either of the two action lists. Then, participants were required to press the left key on the serial response box (Psychology Software Tools, Inc.) to indicate old words and the right key for new words using their index fingers. They had to press the keys as fast and as accurately as they could. In each trial, a fixation point was initially presented for 250 ms, followed by a 250-ms blank screen, and finally the stimulus word, which was presented until the participant responded. The interval between trials was 500 ms. Five filler trials were inserted at beginning of the task. The words used in filler trials were not related to either target or distractor words.

Participants then answered questions about the future simulation task, including feasibility (1, surly infeasible; 7, surly feasible), considering the real schedule (1, not at all; 7, highly considering), vividness (1, not at all; 7, extremely), detailedness (1, not at all; 7, extremely), the effort put into constructing the future event representation (1, not at all; 7, extremely), the workload required for constructing the future event representation (1, not at all; 7, extremely), sense of pre-experience (1, not at all; 7, extremely), emotional valence (1, negative; 4, neutral; 7, positive), emotional intensity (1, very week; 7, very strong), importance of imagined contents (1, not at all; 7, extremely) and importance of next week’s experiment (1, not at all; 7, extremely). Additionally, participants answered two questions for a manipulation check: One asked whether they had imagined anything about the performance task during future simulation, and the other asked participants to confirm which action list they had to perform in next week’s experiment. Finally, experimenters debriefed the participants, informing them that the experiment next week did not exist.

### Results

**Figure 2:**
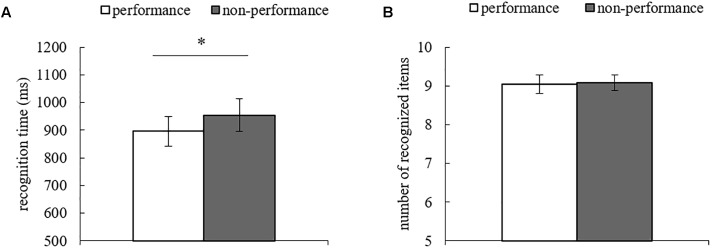
**(A)** Mean correct recognition time and **(B)** mean number of correct recognition items for the performance and non-performance lists in Experiment 1. Error bars indicate standard error. ^∗^*p* < 0.05.

To investigate the activation of concepts related to each action list, we analyzed the recognition time and the number of correct recognition items (recognition performance). A paired *t*-test was conducted to examine differences between list conditions [i.e., the list they were told they would be performing next week (performance condition) and the list they were told they would not be performing (non-performance)]. The mean recognition time for the performance list was significantly faster than that of the non-performance list [*t*(24) = 2.65, *p* = 0.01, Cohen’s *d* = 0.21; Figure 2A]. We observed no significant difference in the mean number of correct recognition items between lists [*t*(24) = 0.14, *p* = 0.89, Cohen’s *d* = 0.04; Figure 2B]. Therefore, the results of recognition time corresponded with our hypothesis.

The mean duration of participants’ future simulation was 181.037 s (*SD* = 25.033). The phenomenological features are shown in Supplementary Table S2. None of the participants reported imagining the actual performance task. Furthermore, all participants correctly answered questions about their assigned performance/non-performance lists. Two of the participants thought about the performance task during the future simulation task, even if only slightly^1^.

### Discussion

The results for recognition time indicated that the concepts of the performance lists were more activated than were those of the non-performance lists, even though participants did not imagine their actual performance on the former lists. In other words, imagining the future events until their intention retrieval opportunity (i.e., the performance of task) led to an intention superiority effect, whereby concepts related to the intended actions were activated, though the opportunity was temporally distant in the future.

This finding has important implications for research on episodic future thinking and prospective memory. Particularly, the findings indicate that spontaneous memory retrieval, which underlies the intention superiority effect, supports episodic future simulation. While there are some studies on the retrieval process for episodic future simulation (Szpunar, 2010; Storm and Jobe, 2012; Giebl et al., 2015; Ditta and Storm, 2016), this is the first to explore the role of spontaneous memory processing in such simulation. This form of processing naturally depends on the mechanism of prospective memory, causing context-sensitive intention superiority effect. Our results further indicate that the activation of intention-related concepts occurs in the context of mentally pre-experiencing future events. Therefore, the present finding extended the role of prospective memory to future simulation.

## Experiment 2 and Experiment 3

In Experiment 1, we found spontaneous activation during future episodic simulation only when participants imagined the events leading up to the retrieval opportunity. Potentially, the same results would occur if participants imagined other future events than those leading up to the retrieval opportunity. To reject this possibility and confirm the context-sensitivity of the intention superiority effect in future simulation, we replicated Experiment 1 while adding a control condition (i.e., thinking of other future events). If the results of Experiment 1 were caused by the context-sensitive intention superiority effect, we could expect the same results in the experimental condition (where participants imagine up to the next experimental session, called the approaching condition)—that is, the conceptual activation of performance list words would be observed in the approaching, but not control condition. In the control condition, participants imagined future events possibly experienced in the following week except the day of the next session while having the same intention as the approaching condition. If the intention superiority effect in the future simulation was context-insensitive and caused by thinking about the future, the effect would be observed in another-day condition. Additionally, we changed the instructions of the recognition task to focus on correct responses to measure accessibility of the concepts related to the studied lists in terms of both recognition time and number. We also replicated Experiment 2 with the same procedure and instruction (Experiment 3), except some environments, to enhance the results’ reliability. Experiment 2 was conducted in Nagoya University, and Experiment 3 in Keio University. The stimulus presentation and data collection were performed using E-prime 2.0 (Psychology Software Tools, Inc.) in Experiment 2, and E-prime 3.0 (Psychology Software Tools, Inc.) in Experiment 3. The serial response box (Psychology Software Tools, Inc.) was used in Experiment 2, and the Chronos (Psychology Software Tools, Inc.) was used as the device to input participants’ response. Here, we report integrated results of the two experiments using meta-analysis.

### Methods

#### Participant

Twenty-three undergraduate students of Nagoya University participated in Experiment 2 (*M*_age_ = 18.87, *SD*_age_ = 0.63), including 10 male and 13 female individuals. One participant who did not believe that the next experiment would actually occur was excluded from the analysis. We also excluded two participants who forgot the performance list. Thus, the data from 20 participants in Experiment 2 (*N*_approaching_ = 11, *N*_another-day_ = 9) were used for meta-analysis. In Experiment 3, 32 undergraduate and graduate students of Keio University participated (*M*_age_ = 20.69, *SD*_age_ = 1.58), including 11 male and 21 female individuals. A participant who forgot the performance list, one who imagined non-specific future events, and one who could not participate in the next week were excluded. Finally, the data from 29 participants in Experiment 3 (*N*_approaching_ = 15, *N*_another-day_ = 14) were used for meta-analysis. The ethics committee of the Graduate School of Environmental Studies at Nagoya University and of the Faculty of Letters at Keio University approved this study. All subjects gave written informed consent in accordance with the Declaration of Helsinki.

#### Procedure

Almost all procedure was the same as in Experiment 1, except the future simulation task. In the future simulation task, participants were randomly assigned to the approaching condition, or another-day condition. Participants of the approaching condition were required to envision specific future events leading up to their arrival at the experimental room, on the day of the experimental session of the following week, as in Experiment 1. Conversely, participants of the another-day condition envisioned events that might occur after school on a day other than that of the next week’s experiment. For this, any day during the week was acceptable. Thus, there were both participants who imagined closer and more distant events than the upcoming experimental day. In the result of such scheduling, the temporal distance of imagined events was substantively random for the next week in both conditions. Participants in another-day condition were asked to press a key to finish the task when they left the school ground. In both conditions, the imagination lasted for the same time as in Experiment 1 (i.e., approximately 3 min, always longer than 2 min). Following this task, they immediately completed the recognition task as in Experiment 1, although with the slightly altered instruction that correctness was more important than speed. Finally, participants answered the same questions about the future simulation task as in Experiment 1. Additionally, participants in Experiment 3 also rated the perspectives during future thinking (1, completely first person; 7, completely third person).

### Results

**Figure 3:**
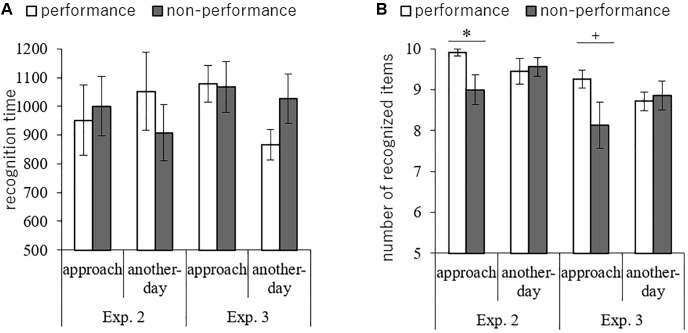
**(A)** Mean correct recognition time and **(B)** mean number of correct recognition items for the performance and non-performance lists in Experiments 2 and 3. Error bars indicate standard error. ^+^*p* < 0.10, ^∗^*p* < 0.05.

To investigate the conceptual activation of the lists, we analyzed the recognition time and number of correctly recognized items. A 2 (condition: approaching, another-day) 2 (list: performance, non-performance) mixed analysis of variance was conducted (condition was between-subjects, list was within-subjects). Regarding the mean recognition time, there was no significant main effect of condition [*F*(1,18) < 0.01, *MSE* = 243733, *p* = 0.98, 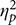 < 0.01] or list [*F*(1,18) = 0.41, *MSE* = 55463, *p* = 0.53, 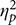 = 0.02], and no interaction [*F*(1,18) = 1.65, *MSE* = 55463, *p* = 0.22, 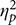 = 0.08; Figure 3A]. For the recognition performance, there was no main effect of condition [*F*(1,18) = 0.02, *MSE* = 1.14, *p* = 0.89, 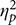 < 0.01] and moderate main effect of list [*F*(1,18) = 3.19, *MSE* = 0.49, *p* = 0.09, 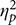 = 0.15]. However, there was a significant interaction between condition and list [*F*(1,18) = 5.21, *MSE* = 0.49, *p* = 0.03, 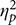 = 0.22]. Then, follow-up comparison revealed that the mean recognition performance of the performance list was significantly greater than was that of the non-performance list in the approaching condition [*t*(10) = 2.47, *p* = 0.03, Cohen’s *d* = 1.32], but there was no significant difference between lists in the another-day condition [*t*(8) = 0.56, *p* = 0.59, Cohen’s *d* = 0.12; Figure 3B]. We conducted the same analysis for Experiment 3. Then, regarding the mean recognition time, there was no significant main effect of condition [*F*(1,27) = 1.79, *MSE* = 130837, *p* = 0.19, 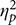 = 0.06] or list [*F*(1,27) = 1.98, *MSE* = 40752, *p* = 0.17, 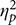 = 0.07], and no interaction [*F*(1,27) = 2.60, *MSE* = 40752, *p* = 0.12, 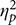 = 0.09; Figure 3A]. For the recognition performance, there was no main effect of condition [*F*(1,27) = 0.04, *MSE* = 2.46, *p* = 0.84, 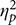 < 0.01] and list [*F*(1,27) = 1.97, *MSE* = 1.80, *p* = 0.17, 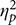 = 0.07]. However, there was a marginally significant interaction between condition and list [*F*(1,27) = 3.27, *MSE* = 1.80, *p* = 0.08, 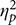 = 0.11]. Then, follow-up comparison revealed that the mean recognition performance of the performance list was significantly greater than was that of the non-performance list in the approaching condition [*t*(14) = 1.80, *p* = 0.09, Cohen’s *d* = 0.89], but there was no significant difference between lists in the another-day condition [*t*(13) = 0.52, *p* = 0.61, Cohen’s *d* = 0.12; Figure 3B]. Thus, the results for recognition performance supported our hypothesis, while the those of recognition time did not.

**Figure 4:**
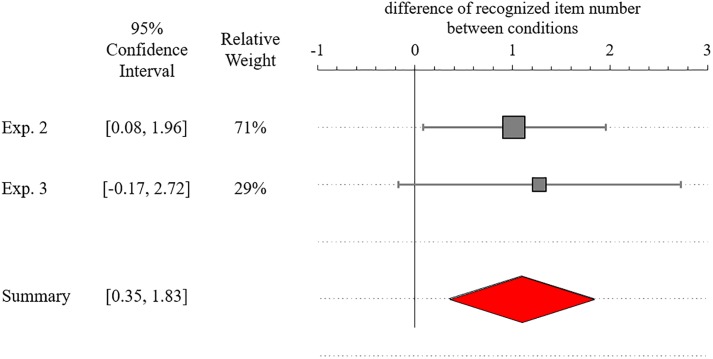
Meta-analysis of Experiment 2 and replication experiment on the difference of recognition accuracy between the performance and non-performance lists. Positive value indicates that intention superiority occurs in approaching condition (error bars and diamonds represent 95% confidence intervals). Relative weights are the percentages contributed by the three studies to the overall meta-analysis. Small standard deviation and large sample size give higher precision, a shorter confidence interval, and greater weight.

Additionally, we calculated the intention superiority effect caused in each condition by subtracting the accuracy for non-performance list from those for performance list (Table 1). We performed a random effect meta-analysis to test the intention superiority effect by future simulation in approaching condition (Figure 4), using the exploratory software for confidence intervals (ESCI; Cumming, 2012). A positive value represents the occurrence of intention superiority effect in approaching condition. The results of meta-analysis indicated the positive value significantly higher than zero [*M*_estimated_ = 1.09, CI (0.35, 1.83), *p* < 0.01]. This estimate enhanced reliability of the result that the intention superiority effect, measured by recognition accuracy, was context-sensitive.

**Table 1:** The mean difference between performance and non-performance lists in each condition and experiment.

	Approaching		Another-day	
	*M*	*SD*	*M*	*SD*
Experiment 2	0.91	1.22	-0.11	0.60
Experiment 3	1.13	2.45	-0.14	1.03

The mean duration of the episodic future simulation of Experiment 2 in the approaching condition was 185.52 s (*SD* = 19.07), while that of the another-day condition was 187.58 s (*SD* = 50.55). The mean duration of the episodic future simulation of Experiment 3 in the approaching condition was 180.11 s (*SD* = 58.68), while that of the another-day condition was 158.13 s (*SD* = 47.41). According to an unpaired *t*-test, a significant difference between these conditions was not found in both experiments [Experiment 2: *t*(18) = 0.13, *p* = 0.90, Cohen’s *d* = 0.05; Experiment 3: *t*(27) = 1.11, *p* = 0.279, Cohen’s *d* = 0.40]. The phenomenological features are indicated in Supplementary Table S3. Only the vividness of future events in the approaching condition was rated significantly higher than was that in another-day condition of Experiment 2 [*t*(18) = 2.85, *p* = 0.01, Cohen’s *d* = 1.23]. The correct rejection times for distractor words and the number of false positive responses in all experiments were shown in Supplementary Table S4 and S5.

### Discussion

In these experiments, although the significant measures were different from Experiment 1, we confirmed that the activations of intention-related concepts were caused by context of the retrieval opportunity in the future simulation. This finding extends the applicability of the context-sensitive intention superiority effect observed in prospective memory (Goschke and Kuhl, 1993; Sellen et al., 1997; Marsh et al., 1998; Schult and Steffens, 2013) to episodic future simulation.

In Experiments 2 and 3, the recognition time results were inconsistent with our expectation. Contrarily, the recognition performance results did—intention-related concepts were activated by envisioning future events leading up to the retrieval opportunity, even though participants did not imagine the actual task to be performed. Therefore, we captured the context-sensitive intention superiority effect in future simulation again, although the measures differed. No doubt the difference was caused by our alteration of the instructions of the recognition task.

There was no difference in the phenomenological characteristics of the imagined future events or the duration of future thinking between the conditions, except for vividness in Experiment 2. The approaching condition required participants to envision future events leading up to their arrival at the experimental room on the day of the experimental session. This setting may clarify to some extent how we construct spatial or visual representations. However, such a result was not found in Experiment 3. Thus, it was not a robust phenomenological feature.

## General Discussion

We investigated whether intention-related concepts become accessible as individuals approach the retrieval opportunity in episodic future simulation. For this, we conducted two experiments developed from a prospective memory task (Schult and Steffens, 2013). In Experiment 1, we demonstrated that the intention superiority effect arises for temporally distant intentions when participants imagined events approaching the retrieval opportunity in episodic future simulation. In Experiments 2 and 3, we replicated the intention superiority effect and ensured that the effect was context-sensitive, as it did not arise in any other future simulation than that revolving around the retrieval opportunity.

We hypothesized that both the recognition time and recognition performance would be higher for the performance than non-performance list. However, in Experiments 1–3, only recognition time and recognition performance, respectively, supported this hypothesis. The differences are attributable to the changed instructions, as noted above: we asked participants of Experiment 2 to focus on accuracy in the recognition task rather than quickness, which naturally would enhance the accuracy of the old/new judgments. Accordingly, the effect size of the difference between the performance and non-performance list words increased considerably (*d* = 1.32). Of course, this increased accuracy had a trade-off with recognition time. These results were reconfirmed by Experiment 3 and meta-analysis of these two experiments. Regardless, these results collectively support the context-sensitivity of the intention superiority effect.

Importantly, activation of the performance-list-related concepts arose without explicit memory retrieval—almost no participant thought about the performance task during the future simulation task, at least according to their subjective reports. Our results also did not change when we excluded the two participants from the analysis who thought about the performance task. Therefore, the activation of intention-related concepts during future simulation may be due to an implicit memory process rather than an explicit, strategic process. These features are consistent with our knowledge of prospective memory (McDaniel and Einstein, 2000). Almost all previous studies on the topic have indicated that episodic simulation is useful as a tool for improving prospective memory performance (Chasteen et al., 2001; Altgassen et al., 2014; Neroni et al., 2014; Kretschmer-Trendowicz et al., 2016; Terrett et al., 2016), suggesting that people remember to implement an intended future action by preliminarily imagining a future situation wherein they perform that action. Those past studies suggest that future simulation contributes to prospective memory performance. Contrarily, our study revealed that the opposite may be true as well: spontaneous activation of intention-related memory, an aspect of prospective memory, supports the efficient retrieval process involved in episodic future thinking. Thus, prospective memory and future simulation appear to mutually support each other. This is the first study that extends the context-sensitive intention superiority effect to the framework of episodic future simulation. This enables us to apply confirmed knowledge and issues in the literature of prospective memory to future simulation study. For instance, although the prospective memory performance is improved by training (Umeda et al., 2006), such training may enhance plausible future simulation.

Moreover, in supporting episodic future simulation, spontaneously remembered intention-related details may also provide constraints for the contents of the simulated future scenario. If intention-related details are not appropriately activated during future simulation, people could construct future scenarios without considering intended actions. In such future simulation, people have an alternative for future behavior. For example, assume that you have a meeting scheduled at 13:30 tomorrow, which is around the time you have lunch. When you imagine the next day’s lunch, you could construct a scenario of having lunch around 13:30 and engage in other tasks after returning to your office, if the spontaneous remembering about the meeting does not work. In this case, where there is inadequate intention-related contextual detail, constructing a future episode almost without constraints is possible. Therefore, context-sensitive intention superiority is important for constructing a plausible future scenario.

The finding that prospective memory supports future simulation accords with previous neuroscientific studies. The ventromedial prefrontal cortex (vmPFC), particularly Brodmann area 10 (BA10), is known to be activate in episodic future thinking and episodic memory (Okuda et al., 2003; Szpunar et al., 2007; Addis et al., 2009; Stawarczyk and D’Argembeau, 2015). The region is also a central area involved in prospective memory (Burgess et al., 2003; Burgess et al., 2007; Hashimoto et al., 2011). Although Okuda et al. (2003) suggested that the activation of BA10 may reflect the processing of future intentions during future thinking, it remains unclear how it relates to episodic simulation. The present study offers a possible suggestion: during episodic simulation, BA10 may be involved in monitoring pre-experience contexts for the future scenario and activating context-related details as the future scenario progresses toward the foreseen retrieval opportunity.

This study has some limitations. First, we cannot exclude the possibility that conceptual activation was caused by “spreading activation” (Yantis and Meyer, 1988) when participants imagined a scene associated with the experimental session. This problem should be investigated by adding a condition requiring participants to imagine a future scenario after the experimental session of the following week or the performance task. Such an experiment would enable us to investigate another important feature of the intention superiority effect—that the intention is deactivated after its completion (Marsh et al., 1998). Second, we did not control the temporal distance of imagined future events from the upcoming experimental session. Participants envisioned the events immediately prior to their arrival at the experimental room; it is possible that intention-related details activate at an earlier point during episodic future simulation. There was a possibility that the imaging temporally proximal events caused the activation of the intention. Clarifying the boundary between activation and deactivation of intention would help us understand the efficiency of future simulation. Relatedly, it would be important to determine whether prospective-memory-like processing also occurs in simulation of “far” future events. The present task required participants to imagine events occurring on one day a week later, making it a relatively near future. Many previous studies have noted changes in phenomenological features and neural activities with temporal distance (D’Argembeau and Van der Linden, 2004; Addis and Schacter, 2008; D’Argembeau et al., 2008). For example, images of near future events tend to be highly detailed, while those of far future events are more abstracted (Trope and Liberman, 2003; D’Argembeau and Van der Linden, 2004, 2006; Trope and Liberman, 2010; Arnold et al., 2011b; D’Argembeau and Van der Linden, 2012). This implies that people use relatively fewer details when envisioning far future scenarios, which may suggest that retrieval processing styles change with temporal distance. In a far future simulation, the context-sensitive intention superiority effect might not arise, or be much weaker, because the far future simulation does not demand many details. If retrieval processing in future simulation changes with temporal distance, such flexible change itself may be an adaptive system. In future studies, we need to investigate these points.

Future simulation is often studied in the context of evolution and adaptation (Atance and O’Neill, 2001; Suddendorf and Corballis, 2007; Suddendorf et al., 2009; Schacter, 2012; Szpunar et al., 2013; Arnold et al., 2016). The present findings have implications for such research. Specifically, intention-related information spontaneously activated ahead of explicitly thinking about that intention in future simulation. Such cognitive processing might cause individuals to think about one future task after another automatically. That is, the spontaneous activation during future simulation would accelerate future thinking. The context-sensitive intention activation should need event/context cognition or recognition of causal relationships between events. Such an idea would intrinsically correspond to the theory of event models (e.g., Radvansky and Zacks, 2014), which also refer to generating event prediction. A wandering mind has prospective bias (Baird et al., 2011; Smallwood et al., 2011), and the acceleration possibly underpins such bias. Given that the functions of future thinking include high-level cognitive activity (i.e., decision-making, action planning, and self-regulation) in daily life (D’Argembeau et al., 2011), the acceleration is also beneficial for adaptation (Moors and De Houwer, 2006).

In summary, we found that, during future simulation, intention-related concepts spontaneously activate as the retrieval opportunity for that intention approaches (i.e., the context-sensitive intention superiority effect). Such conceptual activation ahead of envisioned future events may enable us to construct plausible scenarios in episodic future simulation.

## Data Availability

The datasets generated for this study are available on request to the corresponding author.

## Author Contributions

All authors collaboratively developed the study design, interpreted the results, and approved the final version of the manuscript. YI programmed the computer tasks, collected and analyzed the data, drafted the manuscript, and all other authors provided critical revisions.

## Conflict of Interest Statement

The authors declare that the research was conducted in the absence of any commercial or financial relationships that could be construed as a potential conflict of interest.

## Footnotes

1 The results did not change even after removing these participants from the analysis. The mean recognition time for the performance list was significantly faster than was that of the non-performance list (*t*(22) = 2.25, *p* = 0.04, Cohen’s *d* = 0.19). There was no significant difference between conditions in the mean number of correct recognition items between lists (*t*(22) = 0.14, *p* = 0.89, Cohen’s *d* = 0.04).
