# Supplementary material for: Spontaneous Activation of Event Details in Episodic Future Simulation
Source: Front Psychol. 2019 Mar 21;10:625. doi: 10.3389/fpsyg.2019.00625 (PMC6437097; doi:10.3389/fpsyg.2019.00625)
Supplement: Supplementary file 1 [file Table_1.DOCX]

Supplementary Material

# Supplementary Tables

**Supplementary Table 1.** The stimuli for the recognition task in Japanese and English translation.

| Word Category | |  | Original Stimulus | |  | English Translation | |
| --- | --- | --- | --- | --- | --- | --- | --- |
|  |  |  | noun | verb |  | noun | verb |
| target | brewing coffee |  | フィルター | はめる |  | filter | set |
|  |  |  | 湯 | 注ぐ |  | hot water | pour |
|  |  |  | コーヒー | わける |  | coffee | separate |
|  |  |  | 砂糖 | 溶かす |  | sugar | melt |
|  |  |  | ミルク | 加える |  | milk | add |
|  | making a sandwich | | ベーコン | 焼く |  | bacon | roast |
|  |  |  | レタス | ちぎる |  | lettuce | tear |
|  |  |  | トースト | 作る |  | toast | make |
|  |  |  | マヨネーズ | 塗る |  | mayonnaise | spread |
|  |  |  | 具 | はさむ |  | fillings | sandwich |
| distractor | brewing coffee |  | カップ | 温める |  | cups | warm |
|  |  |  | 粉 | 置く |  | powder | fill |
|  |  |  | ポット | 沸かす |  | kettle | switch-on |
|  | making a sandwich | | ハム | 切る |  | ham | cut |
|  |  |  | 卵 | 乗せる |  | egg | put |
|  |  |  | 野菜 | 刻む |  | vegetables | hash |
|  | unrelated |  | 新聞 | 読む |  | newspaper | read |
|  |  |  | スリッパ | 履く |  | slipper | wear |
|  |  |  | ハンガー | 曲げる |  | hanger | bend |
|  |  |  | ボール | 転がす |  | ball | trundle |

**Supplementary Table 2.** Means and standard deviations of ratings for characteristics of imagined future events in Experiment 1.

| Question items | *M* | *SD* |
| --- | --- | --- |
|  |  |  |
| Feasibility | 6.160 | 0.473 |
| Considering the real schedule | 5.640 | 1.680 |
| Vividness | 4.920 | 1.077 |
| Detail | 4.880 | 1.054 |
| Effort | 5.720 | 0.891 |
| Workload | 4.040 | 1.594 |
| Pre-experience | 4.880 | 1.236 |
| Emotional valence | 4.200 | 0.957 |
| Emotional intensity | 3.520 | 0.963 |
| Importance of imagined contents | 3.960 | 1.338 |
| Importance of next week’s experiment | 4.840 | 1.248 |

**Supplementary Table 3.** Means and standard deviations of ratings for characteristics of imagined future events in Experiment 2 and replication experiment, including results of t-tests of between-condition differences for each variable.

|  | Question items | Approaching | |  | Another-day | |  | *T* | *p* | Cohen’s  *d* |
| --- | --- | --- | --- | --- | --- | --- | --- | --- | --- | --- |
|  |  |  |  |  |  |  |  |  |  |  |
|  |  | *M* | *SD* |  | *M* | *SD* |  |  |  |  |
|  |  |  |  |  |  |  |  |  |  |  |
| Exp. 2 | Feasibility | 6.182 | 0.603 |  | 6.111 | 0.601 |  | 0.261 | .797 | .112 |
|  | Considering the real schedule | 5.273 | 2.240 |  | 5.778 | 1.093 |  | 0.617 | .545 | .266 |
|  | Vividness | 4.727 | 1.679 |  | 2.889 | 1.054 |  | 2.850 | .011 | 1.227 |
|  | Detail | 4.455 | 1.128 |  | 3.889 | 1.269 |  | 1.055 | .305 | .454 |
|  | Effort | 5.455 | 1.293 |  | 5.000 | 1.000 |  | 0.863 | .400 | .371 |
|  | Workload | 4.182 | 1.662 |  | 4.889 | 1.269 |  | 1.048 | .308 | .451 |
|  | Pre-experience | 3.909 | 1.300 |  | 4.333 | 1.118 |  | 0.772 | .450 | .332 |
|  | Emotional valence | 4.455 | 1.128 |  | 4.778 | 1.302 |  | 0.595 | .559 | .256 |
|  | Emotional intensity | 3.545 | 1.508 |  | 3.778 | 1.202 |  | 0.375 | .712 | .161 |
|  | Importance of imagined contents | 3.818 | 0.982 |  | 4.111 | 0.928 |  | 0.680 | .505 | .293 |
|  | Importance of next week’s experiment | 5.091 | 0.831 |  | 4.667 | 1.323 |  | 0.876 | .393 | .377 |
| Exp. 3 | Feasibility | 6.133 | 0.640 |  | 6.071 | 1.072 |  | 0.190 | .850 | .069 |
|  | Considering the real schedule | 5.467 | 2.031 |  | 5.786 | 1.672 |  | 0.460 | .649 | .166 |
|  | Vividness | 4.933 | 1.163 |  | 5.000 | 0.784 |  | 0.180 | .859 | .065 |
|  | Detail | 4.400 | 0.986 |  | 4.929 | 0.616 |  | 1.717 | .097 | .620 |
|  | Effort | 5.933 | 0.961 |  | 5.643 | 1.499 |  | 0.626 | .537 | .226 |
|  | Workload | 4.400 | 1.920 |  | 3.643 | 1.499 |  | 1.178 | .249 | .425 |
|  | Pre-experience | 5.067 | 1.280 |  | 4.786 | 1.369 |  | 0.571 | .573 | .206 |
|  | Emotional valence | 4.333 | 1.447 |  | 4.429 | 0.852 |  | 0.214 | .832 | .077 |
|  | Emotional intensity | 3.600 | 1.595 |  | 3.571 | 1.697 |  | 0.047 | .963 | .017 |
|  | Importance of imagined contents | 3.533 | 1.356 |  | 4.357 | 1.550 |  | 1.526 | .139 | .551 |
|  | Importance of next week’s experiment | 5.133 | 0.743 |  | 5.286 | 1.069 |  | 0.448 | .658 | .162 |
|  | Perspective | 2.267 | 2.463 |  | 2.071 | 1.979 |  | 0.234 | .817 | .085 |

**Supplementary Table 4.** The correct rejection times for distractor words.

|  |  | Category of distractor words | Condition | | | | |
| --- | --- | --- | --- | --- | --- | --- | --- |
|  |  |  | Approaching | |  | Another-day | |
|  |  |  | *M* | *SD* |  | *M* | *SD* |
| Exp. 1 |  | Performance list-related | 1022 | 410 |  | - | - |
|  |  | Non-performance list-related | 1039 | 523 |  | - | - |
|  |  | Unrelated | 772 | 190 |  | - | - |
| Exp. 2 |  | Performance list-related | 1350 | 1030 |  | 984 | 247 |
|  |  | Non-performance list-related | 1073 | 317 |  | 1120 | 358 |
|  |  | Unrelated | 824 | 274 |  | 766 | 171 |
| Exp. 3 |  | Performance list-related | 1493 | 893 |  | 1219 | 885 |
|  |  | Non-performance list-related | 1163 | 187 |  | 1109 | 474 |
|  |  | Unrelated | 905 | 176 |  | 758 | 160 |

**Supplementary Table 5.** The number of false positive responses.

|  |  | Category of distractor words | Condition | | | | |
| --- | --- | --- | --- | --- | --- | --- | --- |
|  |  |  | Approaching | |  | Another-day | |
|  |  |  | *M* | *SD* |  | *M* | *SD* |
| Exp. 1 |  | Performance list-related | 0.280 | 0.891 |  | - | - |
|  |  | Non-performance list-related | 0.240 | 0.663 |  | - | - |
|  |  | Unrelated | 0.080 | 0.277 |  | - | - |
| Exp. 2 |  | Performance list-related | 0.000 | 0.000 |  | 0.556 | 0.882 |
|  |  | Non-performance list-related | 0.273 | 0.905 |  | 0.111 | 0.333 |
|  |  | Unrelated | 0.000 | 0.000 |  | 0.000 | 0.000 |
| Exp. 3 |  | Performance list-related | 0.133 | 0.352 |  | 0.143 | 0.363 |
|  |  | Non-performance list-related | 0.200 | 0.414 |  | 0.143 | 0.363 |
|  |  | Unrelated | 0.000 | 0.000 |  | 0.000 | 0.000 |
